# Assessing elderly walkability to urban parks using mobility analysis and multi-source data: a case study of central Fuzhou, China

**DOI:** 10.1038/s41598-026-41060-x

**Published:** 2026-04-29

**Authors:** Min Wu, Kaige Zheng, Junhong Chen, Jiaxin Zhang, Mingfei Li, Shihang Wu

**Affiliations:** 1 Architecture and Design College, Nanchang University, No. 999, Xuefu Avenue, Honggutan New District, Nanchang, 330031 China; 2 Design Research Institute of Nanchang University, No. 999, Xuefu Avenue, Honggutan New District, Nanchang, 330047 China; 3 Division of Sustainable Energy and Environmental Engineering, Osaka University, 2-1, Yamadaoka, Suita, Osaka 5650871 Japan; 4 Faculty of Social Sciences, University of Southampton, Southampton, UK

**Keywords:** Elderly walkability, Urban parks, Streetscape perception, Semantic segmentation, Age-friendly cities, Engineering, Geography, Geography, Mathematics and computing

## Abstract

Ensuring equitable park access amid rapid urbanization and population ageing is essential. We propose an integrated framework that couples street-network impedance modelling with streetscape visual perception to assess elderly walkability to parks in central Fuzhou, China. A multi-source dataset—street networks, street view images, park polygons and demographic data—was compiled. Physical accessibility was calculated via weighted path impedance, whereas perceived accessibility was estimated by semantic segmentation of images and machine-learning calibration against expert scores. Combining the two yields a composite walkability index that exposes areas where dense networks coexist with poor visual environments and vice versa, delineating green corridors and underserved neighbourhoods. By fusing objective and subjective dimensions, the framework identifies priority zones for age-friendly design and scalable park-planning interventions, and indicates where network repairs should be coupled with streetscape upgrades to reduce elderly green-access inequities, especially in underserved neighbourhoods.

**Supplementary Information:**

The online version contains supplementary material available at 10.1038/s41598-026-41060-x.

## Introduction

Amid the accelerating process of global urbanization, the accessibility, equity, and health-supporting functions of urban green spaces have emerged as critical indicators for assessing urban sustainability and residential quality of life^1^. Walking, as the most fundamental and universally applicable mode of travel, has garnered increasing attention within the domains of urban planning and transport policy. In particular, the walkability of green spaces—such as urban parks and neighborhood green areas—exerts a direct influence on residents’ spatial behavior and their physical and mental well-being^2^.

In the context of rapid population ageing, elderly individuals exhibit a heightened dependence on walking and are increasingly sensitive to changes in their surrounding environments^3,4^. Their mobility outcomes are also shaped by the interaction between environmental demands and individual functional capacities, a relationship long emphasized in environmental gerontology and person–environment fit theory^5,6^. Comparative evidence from dense Asian and European cities similarly shows that older adults’ walking is shaped not only by “can it be reached” but also by perceived supports and barriers along the route—such as shade, legibility, enclosure, and safety cues—consistent with person–environment fit. Recent streetscape-imagery studies further indicate that perception variables (e.g., green view, interface continuity, openness) provide explanatory power beyond network distance in explaining walking willingness and exposure^7,8^. In elderly-focused settings, these perceptual factors often mediate or amplify the effects of physical distance, helping to explain why nominal coverage does not necessarily translate into effective use. This body of evidence substantiates our choice to pair an impedance-based model with a perception component when assessing elderly walkability. High-quality walking accessibility ensures not only the daily convenience and independence of older adults but also their social participation, psychological well-being, and overall quality of life. Thus, it is fundamental to the development of age-friendly green infrastructure^9,10^. Prior studies have noted that urban parks often fail to fully accommodate elderly users, who encounter both physical and social barriers to recreation^11^. Despite recent methodological advances in evaluating walking accessibility, most studies continue to prioritize spatial distance, focusing on the physical connectivity and cost modeling of travel routes. An origin–destination matrix model based on service area buffers and network analysis has been employed to evaluate facility coverage^12^. Slope factors have also been incorporated into traditional shortest path algorithms to simulate the terrain tolerance of elderly individuals^13^. In addition, impedance variables such as traffic signal delays and road width have been integrated into weighted street network systems^14^. While these models effectively estimate whether a destination can be reached, they often overlook environmental experience factors that shape actual walking behavior.

To address this limitation, perceived accessibility frameworks have been increasingly incorporated into recent scholarship, allowing subjective experiences of street environments to be integrated into evaluation systems. Perceptual indicators encompassing street vitality, safety, and comfort have been developed^15^. Visual enclosure and facade diversity have been proposed as proxies for street attractiveness^2^. Moreover, visual greenery and permeability indicators have been extracted from streetscape imagery to preliminarily establish an assessment model for elderly-friendly perceived environments^16^. Moreover, behavioral perspectives suggest that psychological intentions act as mediators between accessibility and actual park use, with attitudes, perceived control, and social norms all significantly influencing walking decisions^17^.

However, most of these methods still rely on surveys and manual scoring, limiting their use in large-scale, multi-site, and cross-scale analyses. Furthermore, there remains an absence of an integrated modeling framework that can effectively bridge perceived and physical accessibility, with limited capacity for translating data from individual paths to service units.

Beyond survey-based frameworks, deep learning–based perception modelling enables city-wide scalability and consistent estimation of walking-related perceptions from streetscape imagery, alleviating small samples, inter-rater variability, and cost constraints typical of traditional ratings. This advantage is particularly relevant in dense Asian cities, where fine-grained blocks and mixed traffic generate pronounced path-level variability that survey-based approaches may inadequately capture, whereas European renewal contexts often benefit from more standardized street conditions^18^.

This contrast motivates our image-based perception component, which employs a pretrained end-to-end VWPCL model calibrated against elderly ratings to retain context-specific validity while gaining coverage and comparability. Semantic segmentation is used only as an auxiliary interpretation tool rather than to generate perception scores.

Accordingly, this study centers on urban parks—the most representative service nodes within urban green spaces—and proposes an integrated framework that couples impedance modeling with a calibrated, segmentation-based perception component, capturing both objective reachability and subjective street-environment evaluations in a single, scalable assessment. Through semantic segmentation of street-view images to extract environmental features, coupled with a weighted path-impedance model, we construct a dual-dimensional assessment system tailored to the elderly population. This framework facilitates path-level analyses at a micro-spatial scale as well as regression analyses at the park scale. Our goal is to identify hidden spatial inequities in green-space access and to provide a systematic framework for assessing, improving, and optimizing age-friendly public spaces.

Behavioral perspectives such as the theory of planned behavior have been applied in mobility contexts, demonstrating that attitudes, perceived control, and social norms jointly predict walking intentions beyond distance measures alone^19,20^. Building on these insights, our framework integrates a perception component with an impedance-based model so that both objective reachability and subjective evaluations of street environments are captured in a single, scalable assessment.

## Related work

### Conceptual foundations and research background of walkability

Walkability serves as a pivotal metric for evaluating the ease with which individuals navigate urban spaces to access desired destinations. It has been widely applied in fields spanning transportation, public health, land use, and green space planning. Fundamentally, walkability encapsulates the intricate interplay among individuals, the environment, and pathways, reflecting not only the spatial configuration and service layout but also the essential requirements of urban equity and daily convenience^21,22^.

Against the backdrop of advancing green mobility and ecological urbanism, walking has emerged as the most fundamental, low-carbon mode of travel, serving as a central element of urban green infrastructure systems. Green spaces—particularly urban parks—function as vital conduits for the public to access ecosystem services and health-related benefits. The walkability of these spaces transcends mere physical access, embodying the social dimension of equitable green space distribution. During periods of rapid urban expansion, vulnerable populations such as the elderly and low-income groups often reside in areas characterized by “green space accessibility deficits,” facing dual constraints posed by uneven spatial distribution and inhospitable walking environments^23^.

These disparities are not merely spatial but also functional for older adults. Limited mobility, chronic conditions, and tighter time budgets raise the effective thresholds for “usable” access even where nominal coverage exists. In many inner-city settings, fragmented street connectivity, steep segments, and insufficient shading inflate the perceived and energetic costs of reaching parks, compounding disadvantages for elderly and low-income residents. Consequently, an equity-oriented lens must interrogate both distance/impedance and street-level experience to explain why coverage fails to translate into use for specific subgroups.

Accordingly, the accessibility of green spaces must consider not only physical proximity but also the actual capacity of diverse groups to perceive and utilize such spaces with explicit attention to underserved neighbourhoods and between-group disparities^24^. For older adults, walking remains the most prevalent mode of travel. However, age-related declines in physical capacity, coupled with heightened environmental sensitivities, render factors such as pathway conditions, pedestrian safety, and shaded comfort particularly salient^25^. Traditional models that focus solely on spatial connectivity frequently exhibit a disconnect between “technically feasible routes” and actual walking behaviors—where routes deemed accessible by models remain unattractive to users. From an equity perspective, the development of a dual-dimensional walkability assessment framework—integrating both physical and perceptual dimensions—is imperative for disentangling disparities in green space service provision and informing precision-targeted spatial interventions.

**Fig. 1:**
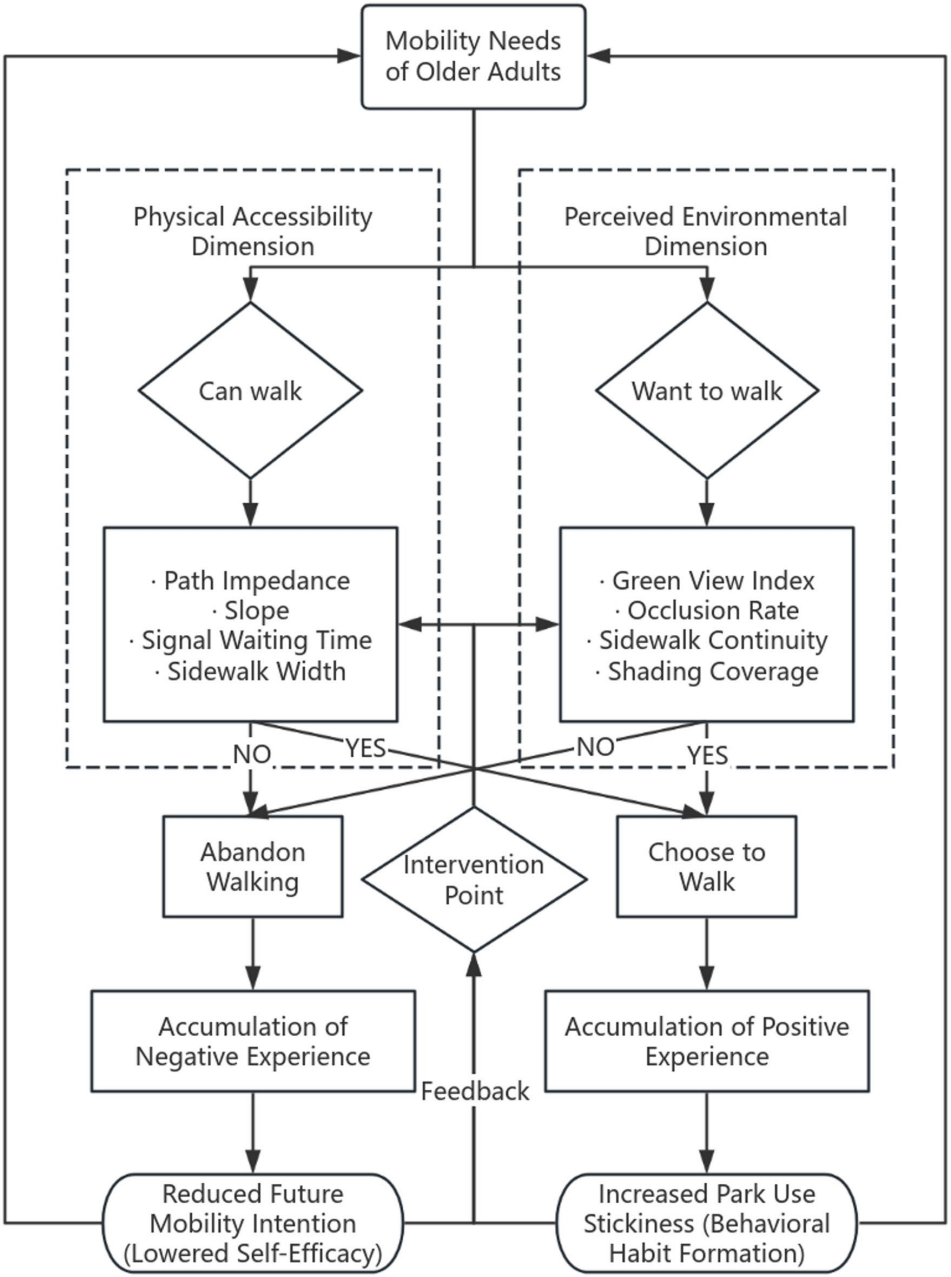
Walking decision-making process of older adults.

To link the aforementioned theoretical concepts with real-world behavioral logic, this section concludes with a walking decision-making flowchart for older adults (see Fig. 1), illustrating how they make walking choices after evaluating both physical accessibility and perceived environment.

### Advances in methodologies for measuring walkability and park accessibility

Existing methods for assessing walkability generally fall into two primary categories: those emphasizing physical accessibility—centered on “can it be reached”—and those exploring perceptual experiences—centered on “is one willing to walk.” These two approaches, rooted in spatial structure and behavioral psychology respectively, are increasingly converging in recent research.

Physical accessibility analyses typically quantify travel costs using metrics such as Euclidean distance, network-based shortest paths, or impedance-weighted route modeling. Traditional approaches—such as Euclidean buffers, least-cost paths, and weighted impedance models—are widely utilized in defining service areas and optimizing facility distributions. Michau (2019) pioneered a network-based OD cost matrix model extensively applied in evaluating urban services such as healthcare and parks^12^. Subsequent research has progressively enhanced the complexity of network models by incorporating impedance factors such as slope^13^, intersection complexity, and signal delays^14^ to more accurately simulate variations in mobility preferences and capacities among different population groups. Notably, weighted path models have proven particularly effective in elderly-focused studies, capturing sensitivities to slope, traffic signals, and sidewalk dimensions, thereby delineating the spatial boundaries of their travel behaviors.

Nevertheless, approaches that rely solely on physical accessibility are inherently limited in capturing the influence of environmental perception on walking behavior. In real-world contexts, visual and psychological elements—such as shading, noise levels, greenery, and pathway coherence—often exert decisive impacts on individuals’ willingness to walk.

Consequently, perceived accessibility research has emerged as a vital complement within the urban green space domain. This line of inquiry foregrounds subjective reactions to the streetscape, aiming to construct evaluation frameworks that more closely align with actual travel psychology. Early studies predominantly employed surveys, interviews, and on-site observations to capture perceptual dimensions such as safety, comfort, vibrancy, and enjoyment. Qin et al. (2025) introduced the Urban Design Quality Index, operationalizing perceptual variables such as visual connectivity, facade transparency, street proportions, and interface vitality to enable quantification of perceptual experiences^15^. Zhang et al. (2025) further emphasized the influence of interface continuity and environmental complexity on walking intentions, advancing methods for quantifying “street attractiveness”^2^.

However, due to high data collection costs and inherent subjectivity in scoring standards, these methods often face constraints in large-scale applications and struggle to establish effective linkages between route continuity and service area scales. The advent of artificial intelligence and computer vision technologies has introduced streetscape imagery as an emergent data source for perceived accessibility analyses, propelling the field into an algorithm-driven era. The Place Pulse project by Quercia et al., for example, leveraged crowdsourced ratings of street-view images to construct “perception maps,” visualizing attributes such as safety, aesthetic appeal, and vibrancy at the city scale.

More critically, advances in deep learning and semantic segmentation have endowed imagery with structured computational capabilities. Li et al. (2022) employed the DeepLabv3+ model for pixel-level semantic segmentation of streetscape images, extracting spatial proportions of urban elements such as vegetation, sidewalks, vehicles, and buildings to generate multidimensional visual metrics—namely, green view index, vehicle obstruction rate, and sidewalk continuity^16^. This approach circumvents the scale, efficiency, and consistency limitations of traditional surveys, offering broader applicability for perceptual assessment. In dense Asian settings with fine-grained blocks and mixed traffic, these indicators enable path-resolution comparisons and reveal distributional disparities that survey-only designs may miss, while European street-renewal cases highlight transferable levers such as moderating enclosure and ensuring sidewalk continuity.

Nonetheless, even algorithm-driven studies remain largely confined to street segments or static points, lacking the capacity to continuously model complete paths or to effectively map route-level perceptual attributes to service-unit scales such as parks. Crucially, an integrated modeling logic linking streetscape perceptual variables with path impedance factors remains absent, often resulting in fragmented analyses and limited explanatory power for behavioral predictions, and leaving the mapping from route-level attributes to park service units under-specified.

In light of the dual challenges posed by spatial equity in green space access and the construction of age-friendly urban environments, there is an urgent need for a multi-scale assessment framework that integrates streetscape perception and route-based accessibility, bridging micro-scale experiential factors with macro-scale service coverage. This study addresses this research gap by proposing a dual-dimensional accessibility framework based on streetscape semantic segmentation and weighted path modeling, offering an analytically robust and operationally feasible tool for optimizing green space equity and walking experience.

In recent years, with the development of multi-source geospatial data and computational modeling tools, walking accessibility research has gradually evolved from traditional geometric buffering or simple network analysis to more comprehensive frameworks that integrate physical structures and subjective perceptions. Zhang et al. (2025) constructed a multi-modal accessibility evaluation system based on OpenStreetMap network structures and street view images, incorporating path topology indicators and visual perception features (e.g., green view index, openness), and introduced machine learning algorithms to extract street-level scores for spatial exposure and safety perception^2^. This approach not only reveals how street structures influence mobility behavior but also emphasizes the interaction between “physical accessibility” and "perceived accessibility," providing important insights for the daily travel scenarios of elderly populations.

This study provides a methodological reference for the present paper: In the walking accessibility framework constructed in this study, both "spatial path structure" and "environmental visual characteristics" are treated as two core dimensions. The physical accessibility and perceived accessibility scores are calculated separately, and their integrated performance and interrelationship are explored through zone overlay and spatial mapping. Compared to traditional single-dimensional modeling, this study attempts to establish an evaluation model that is closer to real-world perceptual experiences, aiming to support more precise configurations of age-friendly green spaces.

### Research positioning and innovations of this study

Looking at existing literature, although significant progress has been made in the theory and methodology of walking accessibility research, there are still certain shortcomings in the areas of evaluation dimensions, scale integration, and group differences. Particularly in the context of building age-friendly cities, traditional studies have mostly focused on physical accessibility measurement, neglecting the heightened sensitivity of elderly individuals to environmental perception during walking^25^. Additionally, while some studies have attempted to incorporate the perceptual dimension, most have relied on subjective questionnaires or single visual indicators, and have yet to form a systematic visual perception modeling approach^26^.

Furthermore, in terms of spatial scale handling, existing studies typically use parks as the analytical unit, applying buffer zone methods to assess service levels. There has been less focus on starting from street-level data points, conducting micro-scale accessibility and perceptual analyses, and then aggregating these back to the park scale. This process of progressively integrating “pedestrian path—perceptual experience—destination” is a more practical approach to understanding the effectiveness of urban green space services in line with real-world walking constraints.

**Fig. 2:**
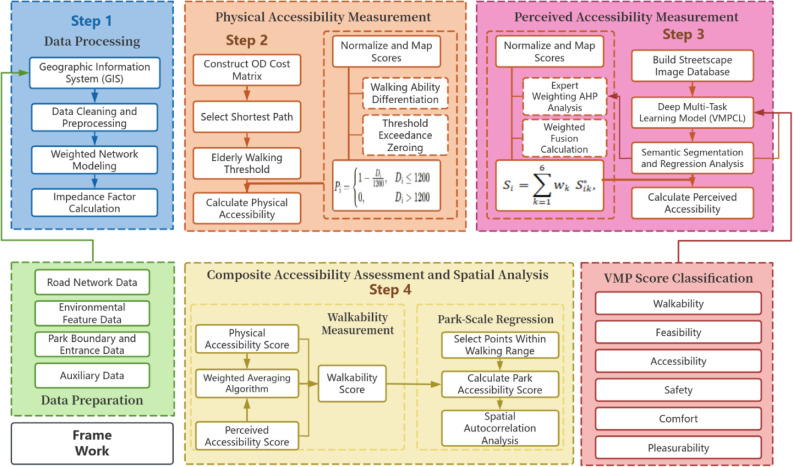
Framework of the study.

Based on the above gaps, this study attempts to expand and innovate in the following three areas:

1. Dual-Dimensional Integrated Walking Accessibility Evaluation Framework: Traditional walking accessibility evaluation methods usually focus on either physical accessibility or perceived accessibility. However, this study combines both, offering a more comprehensive evaluation framework for walking accessibility to age-friendly urban parks by considering both physical accessibility and visual perception. By quantifying the walking distance and visual perception of urban parks from multiple dimensions, it provides a more precise reflection of the walking needs of elderly populations (Fig. 2).
2. Walking Distance-Based Accessibility Measurement Method: Unlike traditional accessibility measurement methods based on buffer zones, this study innovatively introduces walking distance as a measurement criterion. This approach aligns better with the actual travel behavior of elderly populations, providing a more realistic reflection of their actual experiences during walking. By using this method, we avoid common model errors associated with buffer zone methods and provide more accurate data.
3. Multi-Scale Analysis and Path Regression: In contrast to traditional research which analyzes accessibility at the park scale, this study starts from street-level data points and uses a weighted path model to regress from micro-scale street paths to park-scale accessibility. This method not only better aligns with actual walking path experiences, but also reveals the impact of different paths on elderly walking behavior, filling the gap between path scale and service unit scale in existing research^27^.

## Methodology

### Study area

**Fig. 3:**
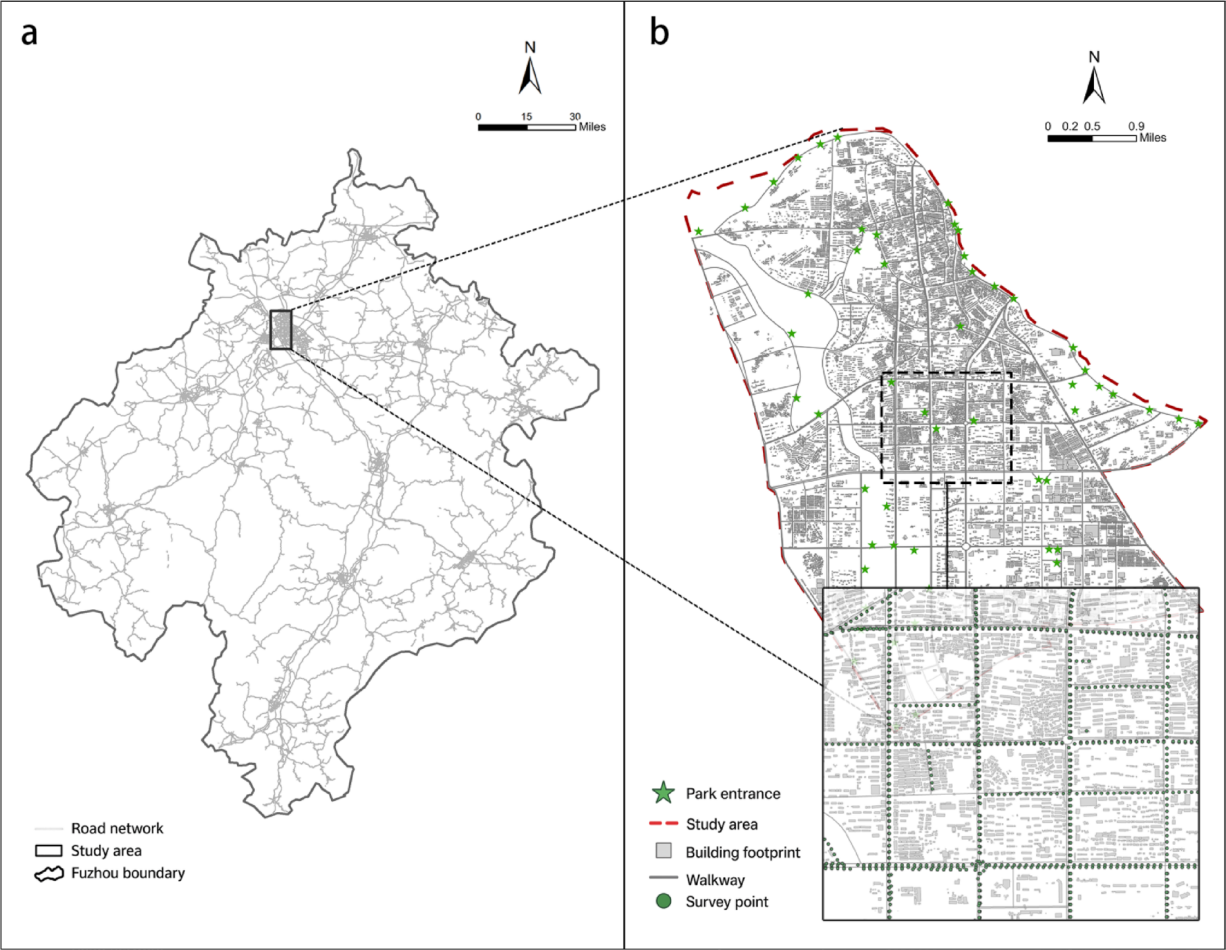
Study Area. The map was created by the authors using ArcGIS Pro (version 3.4.2) (https://www.esri.com/en-us/arcgis/products/arcgis-pro/overview) and finalized in Adobe Photoshop (version 2025) (https://www.adobe.com/products/photoshop.html).

Fuzhou, located in eastern Jiangxi Province, is a national historical city known as the “hometown of talents and culture”. This study selects the central urban area of Fuzhou as its focal region, encompassing the primary built-up areas of the city (see Fig. 3). The study area extends along the Fu River, forming a linear spatial pattern characterized by dense green infrastructure, complex demographic structures, and a prototypical walking network—rendering it an ideal context for research on age-friendly environments. Spatially, the city exhibits a distinctive morphology described as “a central core with two wings, embedded within a landscape of mountains and rivers.”

The selection of this area as the research focus is grounded in three principal considerations:

1. Pronounced ageing Demographics:
  According to the latest census, individuals aged 60 and above constitute 19.7% of the central urban population—markedly higher than the national average of 18.7%. This demographic pattern calls for greater attention to the layout of age-friendly parks, as green space accessibility directly affects the well-being and mobility of older adults^28^.
2. Uneven Distribution of Green Spaces:
  Despite the establishment of over 20 urban parks of varying sizes in planning terms, green space distribution reveals a paradoxical pattern—sparse provision in central areas but dense clustering at the periphery. Additionally, significant disparities in park sizes are observed. In older districts, per capita park space remains acutely insufficient, while the accessibility of riverfront and large-scale parks is often impeded by fragmented road networks. The problem of “visible but inaccessible green space’”is especially pronounced^29^.
3. Heterogeneity in Walking Environment Quality:
  Marked variations exist in the quality of walking environments across neighborhoods. In older districts, pedestrian sidewalks are frequently narrow—often less than 1.2 m wide—with insufficient shading and densely placed traffic signals, all of which diminish walking comfort. These factors reduce the perceived friendliness of streets and diminish park walkability, complicating efforts to evaluate accessibility accurately^28^.

### Data sources

This study establishes a multi-source spatial data system centered on street networks, streetscape imagery, and park entrances. The dataset integrates four components—network structure, visual imagery, spatial boundaries, and demographic context—to support micro-scale accessibility analysis and perception-based evaluation (see Fig. 2).

1. Street Network Data:
  Pedestrian street network data were obtained from OpenStreetMap and manually cleaned to remove non-walkable links, including expressways, closed sections, and vehicle-only ramps. The network was reconstructed in a GIS environment to ensure pedestrian route continuity, with pedestrian overpasses and underpasses connected to ground-level nodes to better reflect actual walking behavior among older adults.
2. Streetscape Imagery and Visual Indicators:
  A 50-m grid sampling scheme was applied across the study area, yielding 5,443 streetscape sampling points. Using the Google Street View API, four-directional images were extracted for each point, resulting in a dataset comprising more than 22,000 images. These images served as the visual input for the perception-based accessibility assessment described in the Methods section.
3. Park Boundaries and Entrance Points:
  Park boundaries and accessible entrances for 18 urban parks were delineated through a combination of the Fuzhou Urban Green Space System Plan, high resolution remote sensing imagery, and points-of-interest (POI) data. A total of 42 park entrances were identified and further verified through streetscape imagery and field checks to ensure consistency between modeled routes and actual walking destinations, thereby avoiding centroid-related distance bias in large parks.
4. Auxiliary Spatial Attributes:
  Building footprints and POI distributions at the street level were incorporated to assess residential density and functional mix. Additionally, elderly population density estimates were integrated to support subsequent analyses of accessibility equity and spatial sensitivity.

### Physical accessibility measurement model

To quantitatively evaluate the objective ease with which elderly individuals can access urban parks, this study developed a network-based physical accessibility model grounded in pedestrian route continuity and distance-based impedance. The model focuses on accurately representing real walking paths rather than simplifying accessibility through Euclidean buffers or centroid-based approximations.

#### Construction of a pedestrian-oriented street network

The pedestrian network was constructed with ArcGIS based on OpenStreetMap road data and refined through manual verification to ensure walkability. Non-pedestrian facilities, such as expressways, highway ramps, and restricted segments, were excluded. Pedestrian overpasses and underpasses were topologically connected to ground-level nodes to preserve route continuity and reflect the actual walking practices of elderly pedestrians.

Unlike coarse buffer-based approaches, this refined network explicitly captures street-level connectivity, intersection structure, and path availability, providing a detailed spatial foundation for elderly-oriented accessibility assessment^30,31^.

#### Estimation of minimum walking distance

Using the reconstructed pedestrian network, we calculated the minimum walking distance between each streetscape sampling point and the nearest park entrance. A total of 5,443 sampling points were treated as origins, and 29 verified park entrances were treated as destinations. Distance calculations were performed using the OD Cost Matrix tool in ArcGIS, which enumerates all feasible walking paths and retains only the route with the shortest cumulative network distance^12^.

For each sampling point $i$, the minimum distance ${\mathrm{D}}_{\mathrm{i}}$ was defined as:

1 $$D_{i} = \min \left( {\mathop \sum \limits_{{e \in p_{ij} }} w_{e} } \right)$$

where ${\mathrm{p}}_{\mathrm{ij}}$ denotes a candidate walking path between sampling point $i$ and park entrance $j$, and ${\mathrm{w}}_{\mathrm{e}}$ represents the effective walking impedance distance of street segment e.

The accumulated walking impedance and its minimization are measured in meters-equivalent (m). To avoid selecting routes that are theoretically optimal but practically inaccessible, we enabled the "Prefer Accessible Path" parameter, which prioritizes routes with a minimum width of 1.5 m and no stairs. Additionally, we set park entrances—rather than geometric centroids—as destination points, for two primary reasons: First, elderly individuals generally terminate their journeys at gates or main access points. Using geometric centroids risks directing routes to inaccessible areas such as lakes or dense forest interiors, resulting in inflated distances of tens or even hundreds of meters. Second, within Fuzhou’s central urban area, the walking distance from the centroid to actual park entrances can reach up to 240 m for the largest parks. Previous studies have shown that intra-park walking exceeding 50 m significantly reduces elderly visitation willingness. All entrance locations were initially verified via satellite imagery, then cross-checked using streetscape images and confirmed through field investigations to ensure positional accuracy within acceptable margins^32,33^.

#### Distance-based accessibility scoring

To enable intuitive spatial comparison and subsequent integration with perceived accessibility, the minimum walking distance from each sampling point to the nearest park entrance was linearly transformed into a dimensionless physical accessibility score. Specifically, the minimum distance ${\mathrm{D}}_{\mathrm{i}}$ (in meters) was mapped onto a standardized range of [0, 1], yielding the physical accessibility score ${\mathrm{P}}_{\mathrm{i}}$.

Previous studies have shown that walking distance strongly constrains park visitation, with visitation probability declining rapidly beyond certain thresholds, particularly among elderly pedestrians. In Chinese urban planning practice, the “15-min life circle” is widely regarded as a comfortable walking range, whereas longer durations typically exceed routine travel willingness. Building on this distinction, the present study adopts an upper-bound threshold to represent the maximum distance elderly individuals are generally willing to walk to parks^2^.

Empirical evidence suggests that the average walking speed of older adults ranges between approximately 0.97 and 1.0 m/s. For simplicity and consistency, a rounded value of 1.0 m/s was adopted, implying that a 20-min walk corresponds to a distance of about 1,200 m. This distance is therefore interpreted as a tolerance limit rather than a comfortable access range^33^.

Based on this, we applied a piecewise linear decay function to calculate accessibility scores:

2 $$P_{i} = \left\{ {\begin{array}{*{20}l} {1 - \frac{{D_{i} }}{1200},} \hfill & {D_{i} \le 1200} \hfill \\ {0,} \hfill & {D_{i} > 1200} \hfill \\ \end{array} } \right.$$

Under this formulation, accessibility decreases linearly with distance and drops to zero beyond 1,200 m. For reference, a distance of 400 m yields a score of 0.67, corresponding to approximately a five-minute slow walk. Sensitivity analyses using alternative thresholds (900 m and 1,500 m) produced highly consistent spatial patterns (Pearson’s r > 0.9; see Supplementary Table 1), indicating that the selected threshold is robust.

**Fig. 4:**
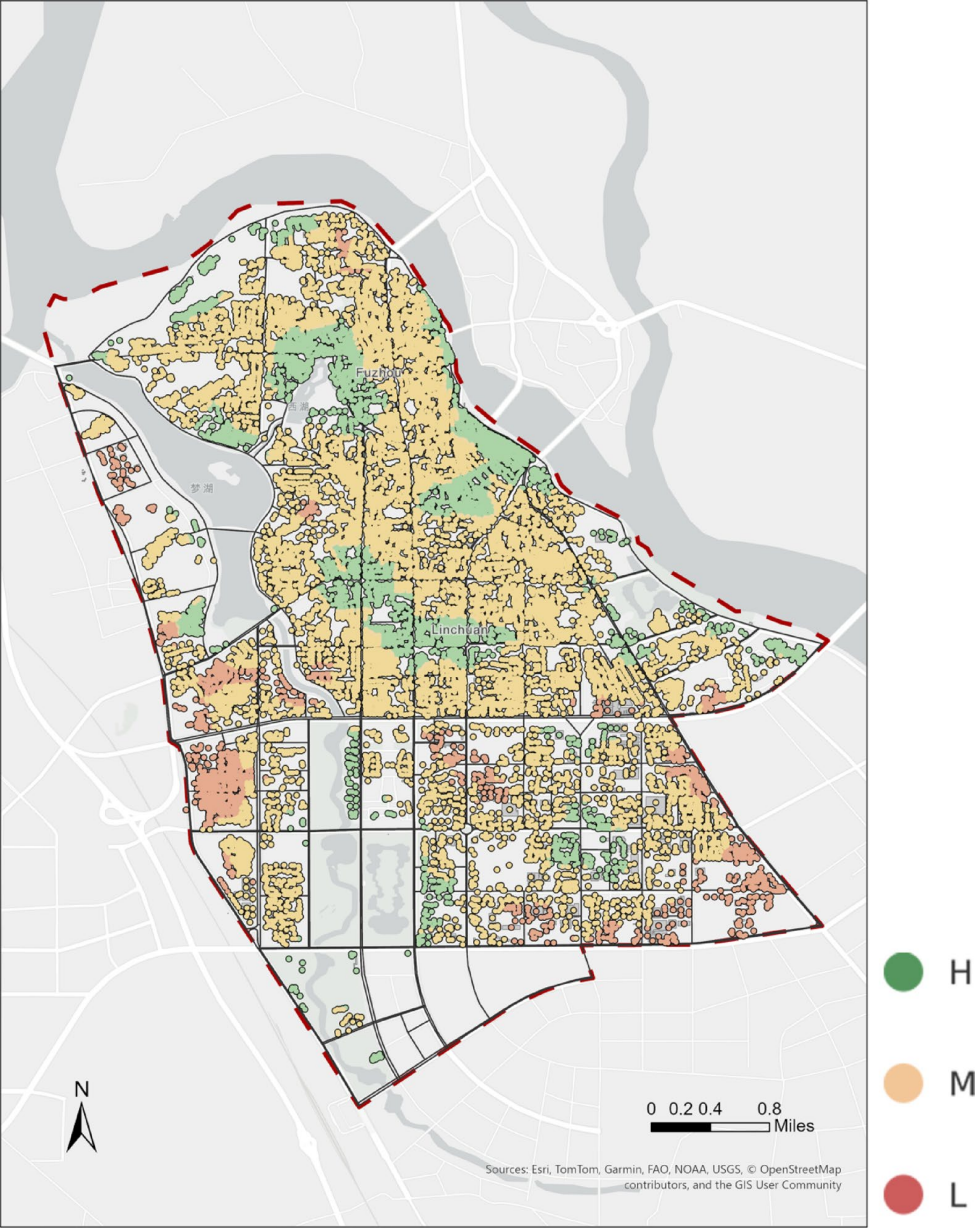
Spatial distribution of residential walkability to urban parks. The map shows physical accessibility scores for residential POIs, calculated based on the minimum walking distance from each residential point to the nearest verified park entrance along the pedestrian network. Scores are normalized to a 0–1 range and classified into high (H), medium (M), and low (L) levels, where higher values indicate shorter effective walking distances and better physical access to parks. The map was created by the authors using ArcGIS Pro (version 3.4.2) (https://www.esri.com/en-us/arcgis/products/arcgis-pro/overview).

Finally, the standardized physical accessibility scores were mapped to residential points of interest to visualize spatial disparities in elderly-oriented park walkability across the study area (Fig. 4).

### Perceived accessibility measurement model

In contrast to physical accessibility, which focuses on whether a destination can be reached, perceived accessibility captures whether elderly individuals are willing to walk, reflecting their subjective evaluation of the street environment^34^. To operationalize this dimension at scale, this study adopts an automated, image-based perception measurement framework that directly translates streetscape imagery into multidimensional perceptual scores relevant to elderly walking experience.

#### image-based prediction of multidimensional walking perceptions

Perceptual accessibility was quantified using a pretrained Visual Walkability Perception Classification Learning (VWPCL) model developed by Li et al.^16^. Unlike conventional approaches that first extract intermediate visual indicators (e.g., pixel proportions) and then estimate perception through regression, the VWPCL model implements an end-to-end prediction strategy that directly maps street-view images to perceptual scores. The model has been trained and validated in prior work using expert-rated immersive street-view data, and is adopted here without retraining.

**Fig. 5:**
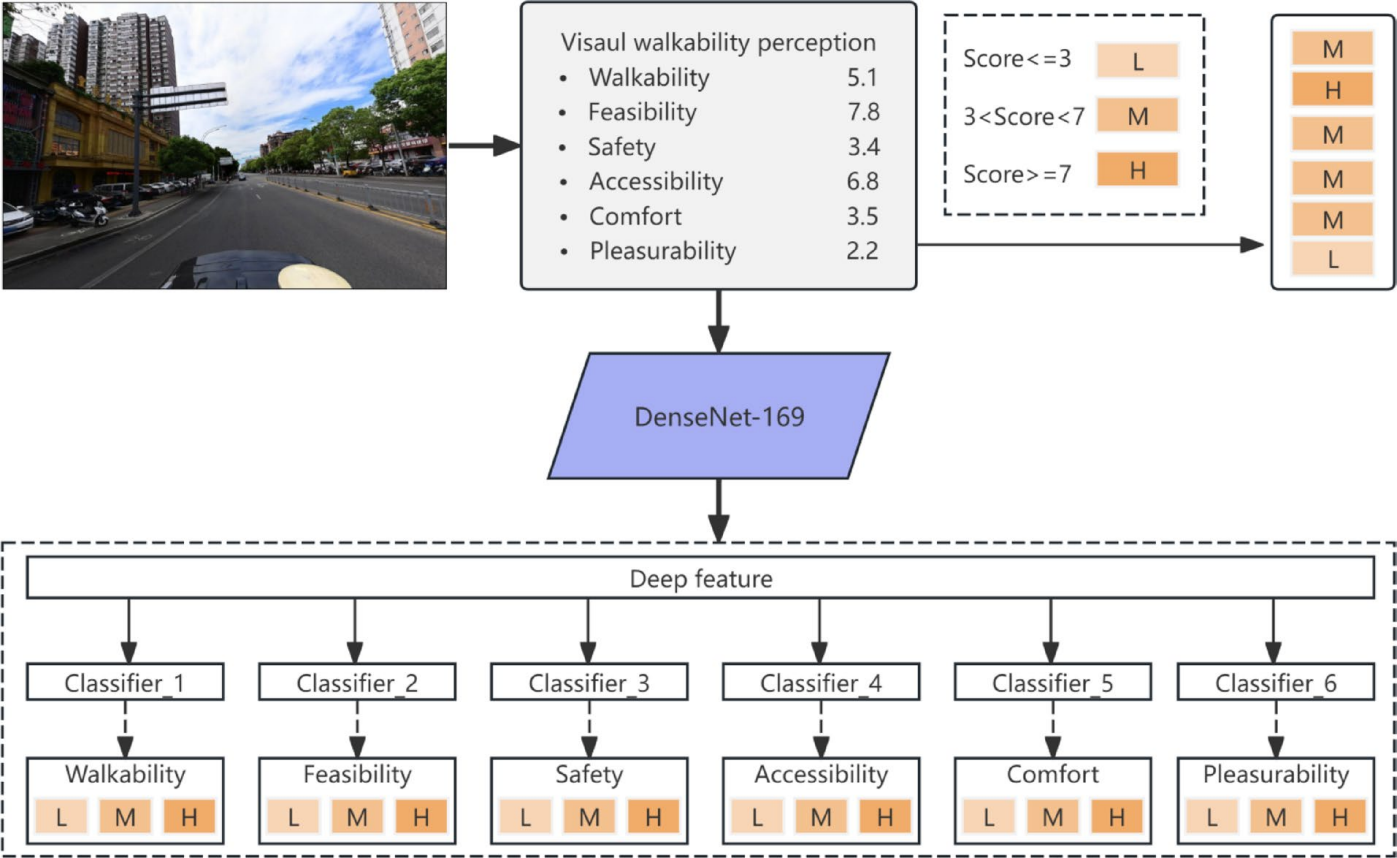
Workflow of the developed VWPCL model. Adapted from Li et al.^16^, with permission under CC BY 4.0.

For each streetscape sampling point, four street-view images corresponding to the cardinal directions (north, east, south, and west) were retrieved. Each directional image was independently processed by the pretrained VWPCL model, which outputs six perceptual scores representing key dimensions of elderly walking experience: walkability, feasibility, accessibility, safety, comfort, and pleasantness (see Fig. 5). The model predicts these scores based on holistic visual patterns in the image, integrating spatial enclosure, greenery structure, surface continuity, and other high-level visual cues learned during prior training^35^.

#### Aggregation of multi-directional perception scores at sampling points

To ensure robustness across varying camera viewpoints and transient visual conditions (e.g., temporary vehicle occlusion or lighting differences), perceptual scores were aggregated across the four directional images at each sampling point. Specifically, for each perceptual dimension, a simple arithmetic mean was calculated across all available directional predictions:

3 $$\overline{v}_{i} = \frac{1}{{\left| {D_{i} } \right|}}\mathop \sum \limits_{{d \in D_{i} }} v_{i,d} ,\quad D_{i} \subseteq \left\{ {N,E,S,W} \right\}.$$

where ${\mathrm{v}}_{\mathrm{i},\mathrm{d}}$ denotes the model-predicted score for direction ddd at sampling point iii, and ${D}_{i}$​ represents the set of valid images available for that point. When fewer than four images were available, aggregation was performed using the existing views. This procedure reduces viewpoint-specific noise while preserving spatial consistency across the study area, resulting in a six-dimensional perceptual score vector for each sampling point.

#### Weighted synthesis of perceived accessibility scores

The six perceptual scores generated by the VWPCL model were originally expressed on a bounded numerical scale. To ensure comparability across dimensions and facilitate integration, all scores were linearly normalized to a unit interval [0,1], preserving relative differences among sampling points while removing scale effects.

To synthesize a single composite perceived accessibility index, the normalized scores were combined using a weighted linear aggregation. The relative importance of the six perceptual dimensions was determined using the Analytic Hierarchy Process (AHP), based on structured expert judgment in age-friendly urban design and planning. The resulting comparison matrix satisfied the consistency criterion (CR < 0.10). Based on the finalized AHP weighting scheme, safety (0.25), comfort (0.20), and accessibility (0.15) were assigned the highest weights and together accounted for approximately 65% of the total contribution, reflecting their primary importance in elderly walking decisions. By contrast, feasibility (0.20), walkability (0.10), and pleasantness (0.10) received comparatively lower weights, representing higher order or supplementary experiential attributes once basic safety and comfort requirements are met.

The composite perceived accessibility score for sampling point $i$ was computed as:

4 $$S_{i} = \mathop \sum \limits_{k = 1}^{6} w_{k} S_{ik}^{*} ,\;\;\;0 \le S_{i} \le 1$$

where ${\mathrm{S}}_{{{\mathrm{ik}}}}^{*}$ is the normalized score for perceptual dimension $k$, and ${\mathrm{w}}_{\mathrm{k}}$ is the corresponding AHP derived weight. Higher values of ${\mathrm{S}}_{\mathrm{i}}$ indicate streetscapes that are visually supportive, safe, and comfortable for elderly pedestrians, whereas lower values indicate environments with perceptual barriers that may discourage walking.

A higher score (approaching 1) indicates a streetscape characterized by abundant greenery, minimal vehicle-induced stress, and continuous, accessible sidewalks, making it visually inviting for elderly pedestrians. Conversely, a lower score—approaching 0—signals environments with significant visual obstruction, lack of greenery, or fragmented walking paths. To validate the spatial differentiation of the results, we assessed between-group differences across distance-to-centroid zones (Core_Q1–Periphery_Q4) using Kruskal–Wallis tests with Bonferroni-adjusted pairwise comparisons.

#### Auxiliary interpretation using semantic segmentation

**Fig. 6:**
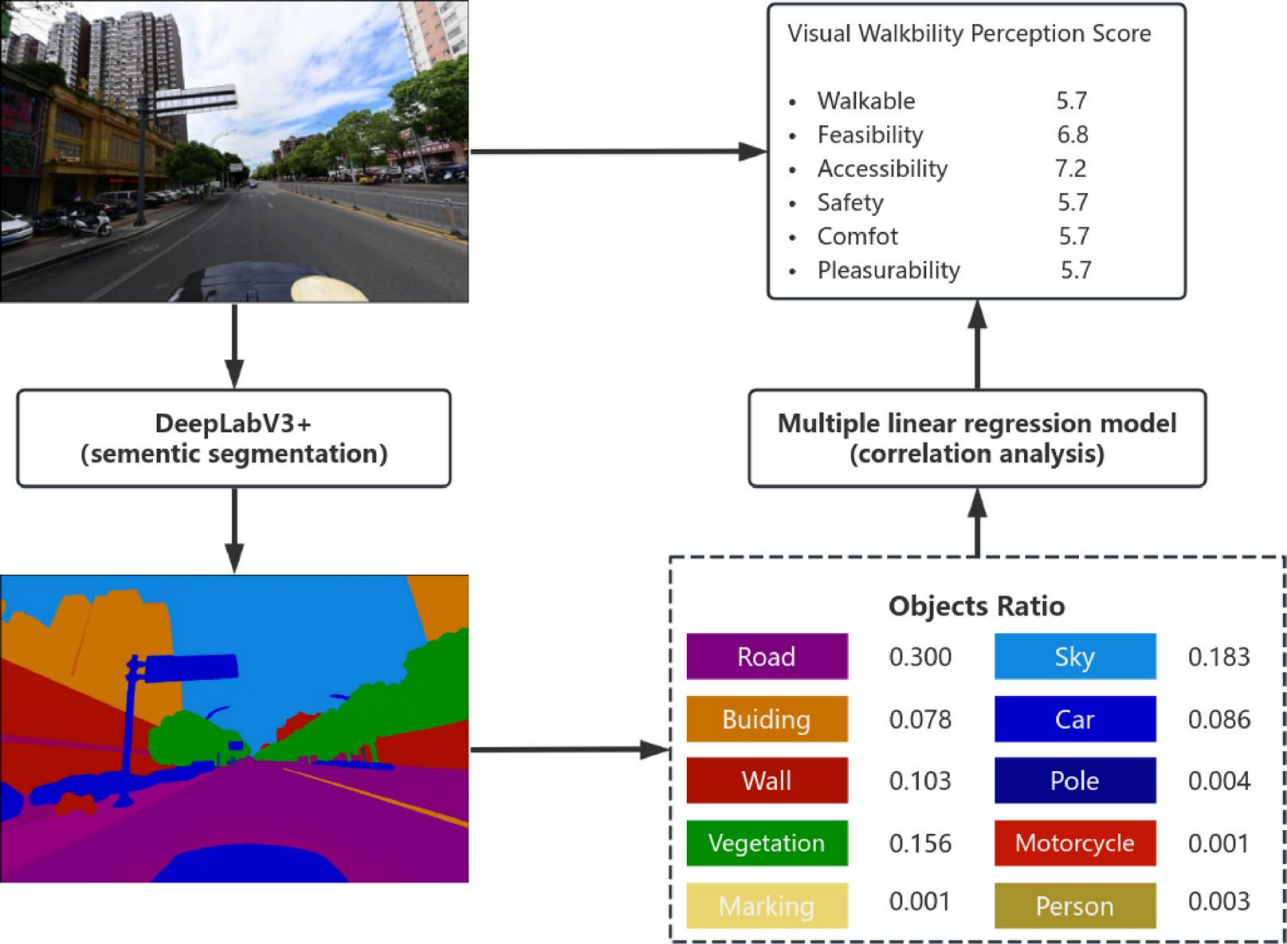
Workflow of semantic segmentation. Adapted from Li et al.^16^, with permission under CC BY 4.0.

To enhance transparency and reproducibility, semantic segmentation was employed as an auxiliary interpretation tool rather than as a core prediction mechanism. All street-view images were processed using a pretrained DeepLabv3+ model to extract pixel-level proportions of key physical elements (e.g., vegetation, sidewalks, vehicles, buildings). These segmentation outputs were used to examine the consistency between model-predicted perceptual scores and observable physical features through descriptive and regression-based analyses (see Fig. 6).

Importantly, semantic segmentation results were not used to generate perceptual scores; instead, they serve to support interpretability and allow independent verification of the environmental attributes associated with different perception dimensions. This two-layer design—end-to-end perception prediction combined with post hoc visual interpretation—ensures both scalability and methodological transparency.

### Composite accessibility measurement model

In the preceding sections, we independently quantified physical accessibility, representing “*whether one can walk*” and perceived accessibility, reflecting “*whether one is willing to walk*.” As previously discussed in Sect.  2, neither dimension alone sufficiently captures the actual walking experience of elderly pedestrians^36^. Solely focusing on physical factors tends to underestimate psychological barriers such as insufficient greenery or visual obstruction, whereas considering perceptual factors alone overlooks objective network-based constraints and spatial impedance^37^.

Therefore, in this section, we integrate both dimensions under a unified 0–1 scale to develop a composite accessibility index ${\mathrm{C}}_{\mathrm{i}}$. using a straightforward linear weighting scheme:

5 $$C_{i} = \beta P_{i} + (1 - \beta )S_{i} ,\;\;0 \le C_{i} \le 1$$

where ${\mathrm{P}}_{\mathrm{i}}$(physical) and ${\mathrm{S}}_{\mathrm{i}}$ (perceived) are standardized scores in [0,1] (dimensionless); $\upbeta$∈ [0,1] is a dimensionless weight; hence the composite ${\mathrm{C}}_{\mathrm{i}}$ also lies in [0,1] and is dimensionless. Here, $\upbeta$ was set to 0.4, assigning weights of 40% and 60% to physical and perceived accessibility, respectively, based on combined theoretical reasoning and empirical robustness checks.

From a theoretical perspective, environmental gerontology, person–environment fit, and the theory of planned behavior emphasize that older adults’ walking intentions are more strongly influenced by perceived safety, comfort, and barrier-free conditions than by pure distance, consistent with the person–environment fit view that supportive cues lower perceived demands while adverse cues raise them.

From a data perspective, perceived accessibility was derived from high-resolution, image-based measurements, whereas physical accessibility was primarily based on network distances and connectivity.

Furthermore, sensitivity analyses confirmed that alternative allocations (50/50, 80/20) yield almost identical spatial patterns (Pearson r > 0.99; Supplementary Table 2). This indicates that the baseline 60/40 weighting scheme is not only theoretically grounded but also empirically robust, offering a balanced representation of elderly pedestrians’ real-world walking experiences. It should also be acknowledged, however, that certain indicators (e.g., shading and greenery) may simultaneously influence both physical impedance and perceived comfort, implying partial dependence between the two dimensions rather than strict independence.

### Validation: internal checks

Internal consistency checks (e.g., distance-threshold sensitivity within 0.9–1.5 km) indicate stable spatial patterns within reasonable bounds (see Supplementary Table 1). External validation using observed walking behavior is beyond the scope of this study and is discussed in the Research Limitations section.

## Results

### Distribution of physical walkability scores

Physical accessibility reflects the objective likelihood that elderly pedestrians can reach parks under the constraints of the existing street network and terrain conditions. Based on the weighted minimum impedance distances, we linearly mapped the results onto a 0–1 scale to derive physical accessibility scores for the 5,443 sampling points. The statistical outcomes are presented in Table 1. The full set of sampling-point accessibility scores is provided in Supplementary Table 3.

**Table 1:** Descriptive statistics of physical accessibility scores.

Statistic	Value
Minimum	0.00
25th percentile	0.18
75th percentile	0.68
Maximum	1.00
Mean	0.43
Standard deviation	0.30
High(H)	0.99
Medium(M)	0.62
Low(L)	0.27

Figure 10 presents the spatial distribution of physical accessibility scores. Both the kernel density estimation plot (Fig. 10a) and the boxplot (Fig. 10b) reveal a right-skewed distribution, with many sampling points concentrated in the moderate-to-low score range.

In the boxplot (Fig. 10b), the median physical accessibility score is 0.43, with an interquartile range (IQR) of 0.18–0.68, covering about half of the observations and indicating substantial variability across neighborhoods. The lowest-scoring quartile (≤ 0.18) is predominantly located where networks are discontinuous, sidewalks are narrow, or routes are circuitous—such as backstreets in the old city center and peripheral streets in newly developed districts. By contrast, the top quartile (≥ 0.68) clusters along direct, well-connected corridors—especially near park entrances and major thoroughfares.

The kernel density plot (Fig. 10a) further shows broad dispersion across the entire score range, underscoring pronounced spatial heterogeneity in physical accessibility.

Figure 4 provides a map-based view of these patterns by classifying residential POIs according to their standardized scores to the nearest park entrance. Using distribution-informed breakpoints (Low ≤ 0.27, Medium 0.27–0.62, High ≥ 0.62; cf. Table 1), high-score clusters emerge along the northern riverfront belt and around several central parks where entrances are dense and routes are direct. Low-score pockets appear toward the south-western and south-eastern edges, where local discontinuities and longer effective walking distances are common. Medium scores dominate the interior grid, indicating moderate access.

Points with a score of 0 fall beyond the 1,200 m walking threshold to the nearest park entrance; although green land may exist in these areas by land-use classification, excessive distance renders them effectively inaccessible on foot. Conversely, points with a score of 1 are concentrated immediately adjacent to entrances, with minimal walking resistance, representing the highest physical accessibility.

Overall, the figures indicate a dispersed pattern with a concentration of moderate-to-low scores and a limited number of high-score pockets, highlighting spatial heterogeneity in physical walkability across the study area.

### Distribution of perceived walkability scores

**Fig. 7:**
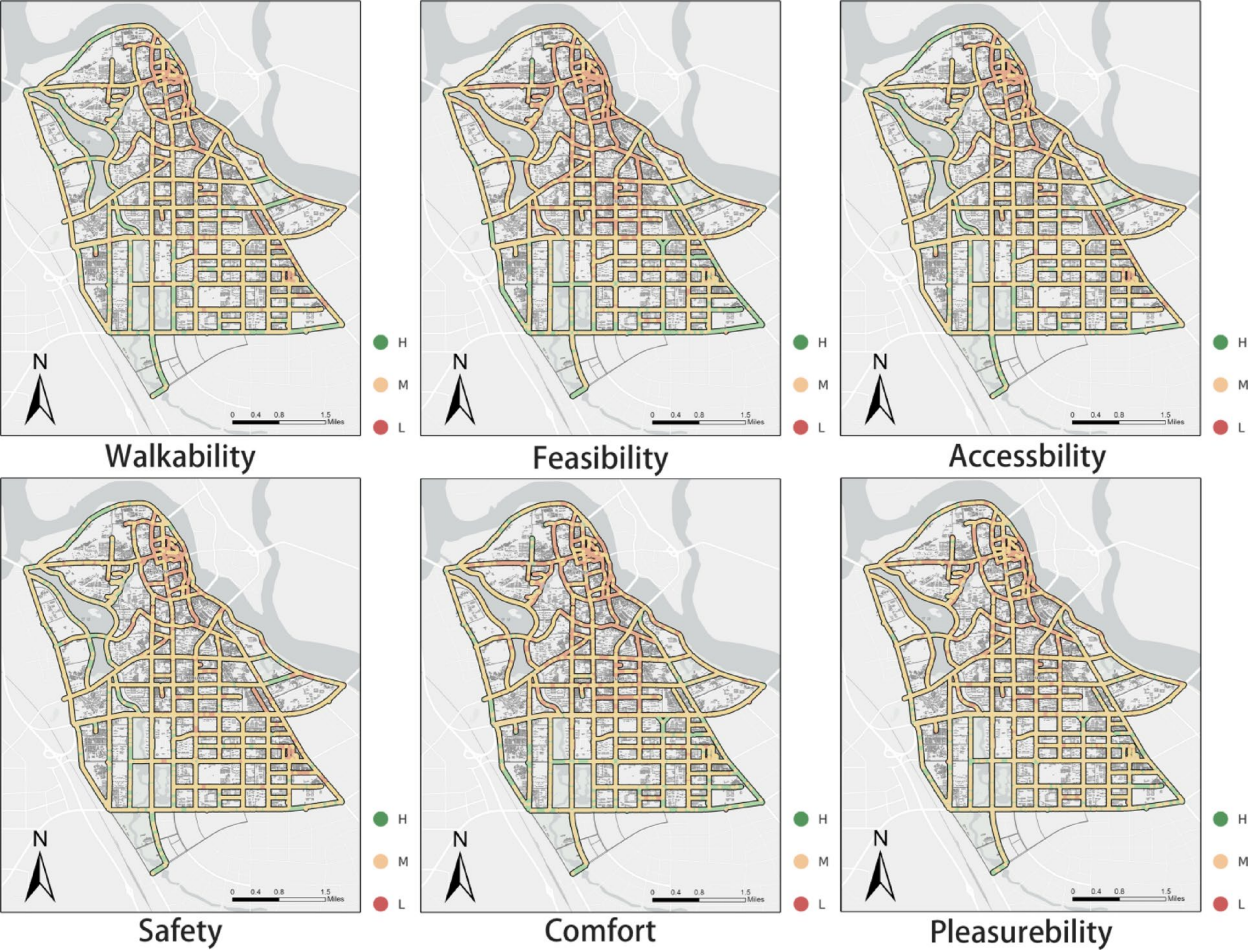
Street-level spatial distribution of six perceived walkability dimensions. This figure presents the classified scores of six perception-based dimensions—walkability, feasibility, accessibility, safety, comfort, and pleasantness—derived at street-level sampling points. Each dimension is grouped into high (H), medium (M), and low (L) categories, allowing cross-dimensional comparison of spatial consistency and divergence in elderly walking perceptions. The map was created by the authors using ArcGIS Pro (version 3.4.2) (https://www.esri.com/en-us/arcgis/products/arcgis-pro/overview).

Perceived accessibility reflects the subjective evaluation of the walking environment by elderly pedestrians. In this study, it was derived through weighted aggregation of multidimensional environmental indicators extracted via semantic segmentation of streetscape images. The spatial distributions of the six perception-based dimensions at the street level reveal both shared spatial cores and localized divergences across the study area (see Fig. 7).The scores were subsequently categorized into High (H), Medium (M), and Low (L) levels using the natural breaks classification method. The statistical outcomes are presented in Table 2.

**Table 2:** Descriptive statistics of perceived accessibility scores.

Statistic	Value
Minimum	0.312
25th percentile	0.458
75th percentile	0.515
Maximum	0.628
Mean	0.487
Standard deviation	0.043
High(H)	0.62
Medium(M)	0.51
Low(L)	0.45

Figure 10 presents the spatial distribution of perceived accessibility scores. Both the kernel density estimation plot (Fig. 10a) and the boxplot (Fig. 10b) demonstrate a right-skewed distribution, with most sampling points concentrated in the lower score range, indicating that the overall visual friendliness of the walking environment remains moderate or suboptimal.

In the boxplot (Fig. 10b), the median perceived accessibility score is 0.487, with an interquartile range (IQR) spanning from 0.458 to 0.515, encompassing approximately 50% of the data points. The relatively narrow box and short whiskers indicate that most scores are tightly clustered within a medium–low range, with relatively few extreme values. The bottom 25% of scores (≤ 0.458) are primarily located in areas characterized by inadequate greenery and poor walking environments, such as narrow alleys in old districts or secluded lanes in newly developed residential areas. Conversely, the top 25% of scores (≥ 0.515) are concentrated near parks and along major green corridors, where ample greenery, open sidewalks, and minimal traffic interference contribute to high visual walkability.

The kernel density plot (Fig. 10a) further confirms this trend, with the highest density of scores appearing in the 0.45 to 0.50 range. This suggests that most urban areas exhibit moderate levels of visual friendliness, with relatively smooth score distributions across the study area. The right-skewed pattern also indicates that while certain locations offer excellent walking environments, the overall visual quality of the streetscape remains modest and warrants further improvement.

Figure 10 further illustrates the spatial distribution of the perceptual scores. In general, higher scores are mostly found along major roads and near parks, while lower scores are concentrated in backstreets and peripheral areas. The contrast between these areas highlights clear spatial disparities in the visual perception of the walking environment.

Overall, the figures indicate that the distribution of perceived scores is relatively concentrated, with a larger proportion of low-scoring areas and more scattered high-scoring areas, reflecting significant differences in the visual perception of the walking environment.

Building on these single-dimension results, we next integrated physical and perceived accessibility into a composite index to capture overall walkability.

### Distribution of composite walkability scores

Following the separate analyses of physical and perceived accessibility, we constructed a composite walkability index by weighting the two dimensions at 60% (perceived accessibility) and 40% (physical accessibility). This integration provides a balanced representation of the overall ease with which elderly residents can access parks. The statistical results are summarized in Table 3, while sensitivity checks are reported in Supplementary Table 2.

**Table 3:** Descriptive statistics of composite accessibility scores.

Statistic	Value
Minimum	0.247
25th percentile	0.359
75th percentile	0.564
Maximum	0.760
Mean	0.465
Standard deviation	0.121
High(H)	0.76
Medium(M)	0.55
Low(L)	0.40

The data in Fig. 10, including the kernel density estimation plot (Fig. 10a) and the boxplot (Fig. 10b), reveal that, unlike the more variable distributions of perceived and physical accessibility scores, the composite accessibility scores exhibit a more balanced and stable distribution. While perceived and physical accessibility scores individually demonstrate large fluctuations—physical scores being broadly dispersed and perceived scores tightly clustered—the composite scores show greater concentration around the middle range due to the compensatory effects of the two dimensions.

Specifically, the median composite score is 0.46, with an interquartile range (IQR) from 0.43 to 0.49, encompassing approximately 50% of all sampling points. This suggests a relatively even score distribution that mitigates extreme volatility inherent in single-dimension assessments.

The observed uniform and stable distribution stems from the complementary nature of the two accessibility dimensions^38^. Areas with high physical accessibility generally feature well-connected walking networks and shorter distances but may suffer from poor greenery or suboptimal environmental comfort, leading to lower perceived scores. Conversely, areas with high perceived accessibility—characterized by pleasant environments and abundant greenery—may compensate for less favorable physical conditions.

Integrating both dimensions balances extreme scores, resulting in moderate composite values across most areas. This demonstrates that the composite index provides a more balanced and realistic evaluation of the walking environment for elderly pedestrians compared to single-dimension metrics.

**Fig. 8:**
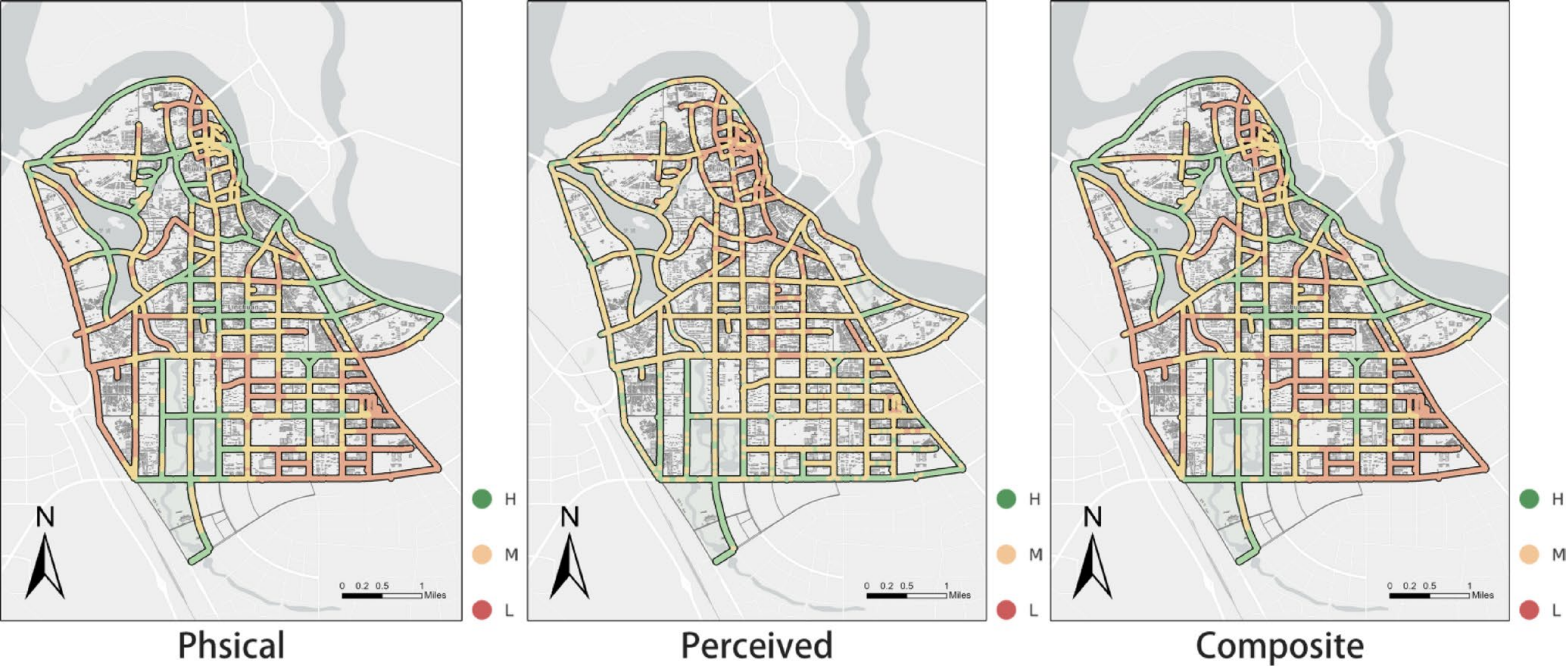
Street-level spatial distribution of physical, perceived, and composite walkability scores. Panels show the classified street-segment scores for (**a**) physical accessibility, (**b**) perceived accessibility, and (**c**) composite walkability, based on street-level sampling points. Scores are grouped into high (H), medium (M), and low (L) categories to highlight spatial contrasts and the compensatory or divergent patterns between objective network-based accessibility and perception-based walkability. The map was created by the authors using ArcGIS Pro (version 3.4.2) (https://www.esri.com/en-us/arcgis/products/arcgis-pro/overview).

A side-by-side comparison of physical, perceived, and composite accessibility highlights pronounced spatial contrasts as well as compensatory patterns across the street network (see in Fig. 8).

### Group differences (core–periphery quartiles)

Across physical, perceived and composite indices, between-group differences are statistically significant (see Supplementary Table 4). Pairwise comparisons indicate that the composite index peaks in the near-core quartile (Q2), whereas perceived scores increase monotonically from Core_Q1 to Periphery_Q4. These patterns corroborate a core–periphery gradient and substantiate the spatial disparities described above.

Interpreted through a person–environment fit lens, the near-core peak (Q2) arises where objective costs are moderate and perceptual burdens are not excessive, whereas higher perceived scores toward the periphery are offset by greater objective costs.

### Composite accessibility and classification analysis at the park scale

The preceding sections evaluated the spatial distributions of physical, perceived, and composite accessibility at the streetscape level within the central urban area. However, from the perspective of urban residents, the ultimate service target is not the individual sampling point but rather the “park” itself. Therefore, in this section, we aggregated the point-based data to the park level, assessing each park’s service performance in terms of both spatial configuration and path-based walking experiences.

Taking the 29 identified park entrances as analytical centers, we employed a network-based analysis approach to extract all sampling points within a 1,200 m walking distance buffer for each park. For each park, we then calculated the average values of physical accessibility, perceived accessibility, and composite accessibility, thus reflecting the park’s actual level of walkability. Detailed park-level scores are listed in Supplementary Table 5.

Based on the composite accessibility scores, coupled with the performance of the three dimensions, we classified the parks into four categories (see Table 4).

**Table 4:** Park classification based on composite accessibility performance.

Category	Classification Criteria	Spatial Implication
Type I (High-High)	${{P}}_{{i}}\ge\bar{{P}},{{S}}_{{i}}\ge\bar{{S}}$	Superior location and favorable environment Priority targets for stability and maintenance
Type II (Perception-Dominated)	${{P}}_{{i}}<\bar{{P}},{{S}}_{{i}}\ge\bar{{S}}$	lightly remote but visually comfortable Suitable for improving path connectivity
Type III (Physical-Dominated)	${{P}}_{{i}}\ge\bar{{P}}, {{S}}_{{i}}<\bar{{S}}$	Convenient access but weak experiential quality Requires greenery enhancement and detail optimization
Type IV (Low-Low)	${{P}}_{{i}}<\bar{{P}}, {{S}}_{{i}}<\bar{{S}}$	Weak in both accessibility and environmental quality In urgent need of systematic intervention and improvement

The park-level patterns underlying this classification are further reflected in Fig. 11. In the physical–perceived accessibility space (Fig. 11a), parks distribute across four quadrants defined by the mean values of the two dimensions, revealing that similar composite scores can arise from different combinations of physical and perceptual conditions. The corresponding bar chart (Fig. 11b) shows that some parks achieve higher composite accessibility mainly through strong physical connectivity, whereas others rely more on favorable perceptual environments to compensate for less advantageous spatial locations. Together, these results highlight the internal heterogeneity of park accessibility and support the typological distinctions summarized in Table 4.

The quadrant classification map clearly illustrates the spatial distribution characteristics of these four park types within the city. Type I parks (High-High) are typically located in the urban core areas; although limited in number, they exhibit a structurally complete layout, benefiting from a synergy of “form-path-perception.” Type III parks (Physical-Dominated) are often small green spaces near major traffic corridors; despite their good physical accessibility, their scores are reduced by insufficient shading, lack of signage, or rugged walking paths.

Type II parks (Perception-Dominated) are generally located in visually appealing but peripheral areas, such as waterfront or campus-like parks, where improving connection routes would effectively expand their service radii. In contrast, Type IV parks (Low-Low) are widely distributed in peripheral zones or older districts, suffering from both structural limitations and poor environmental quality, and thus urgently require systemic upgrades.

### Spatial autocorrelation analysis of composite walkability

In this section, Moran’s I statistic was used to quantify the spatial autocorrelation of composite walkability scores, in order to assess whether areas of high and low walkability exhibit statistically significant spatial clustering or dispersion.

Moran’s I ranges from − 1 to 1. A value near 0 indicates no discernible spatial structure; positive values (I > 0) suggest positive spatial autocorrelation (i.e., clustering), whereas negative values (I < 0) indicate spatial dispersion. Our results reveal a Moran’s I value of 0.391, significantly greater than zero, indicating a moderate 

but significant positive spatial autocorrelation in the distribution of composite walkability scores across the central urban area^39^.

**Fig. 9:**
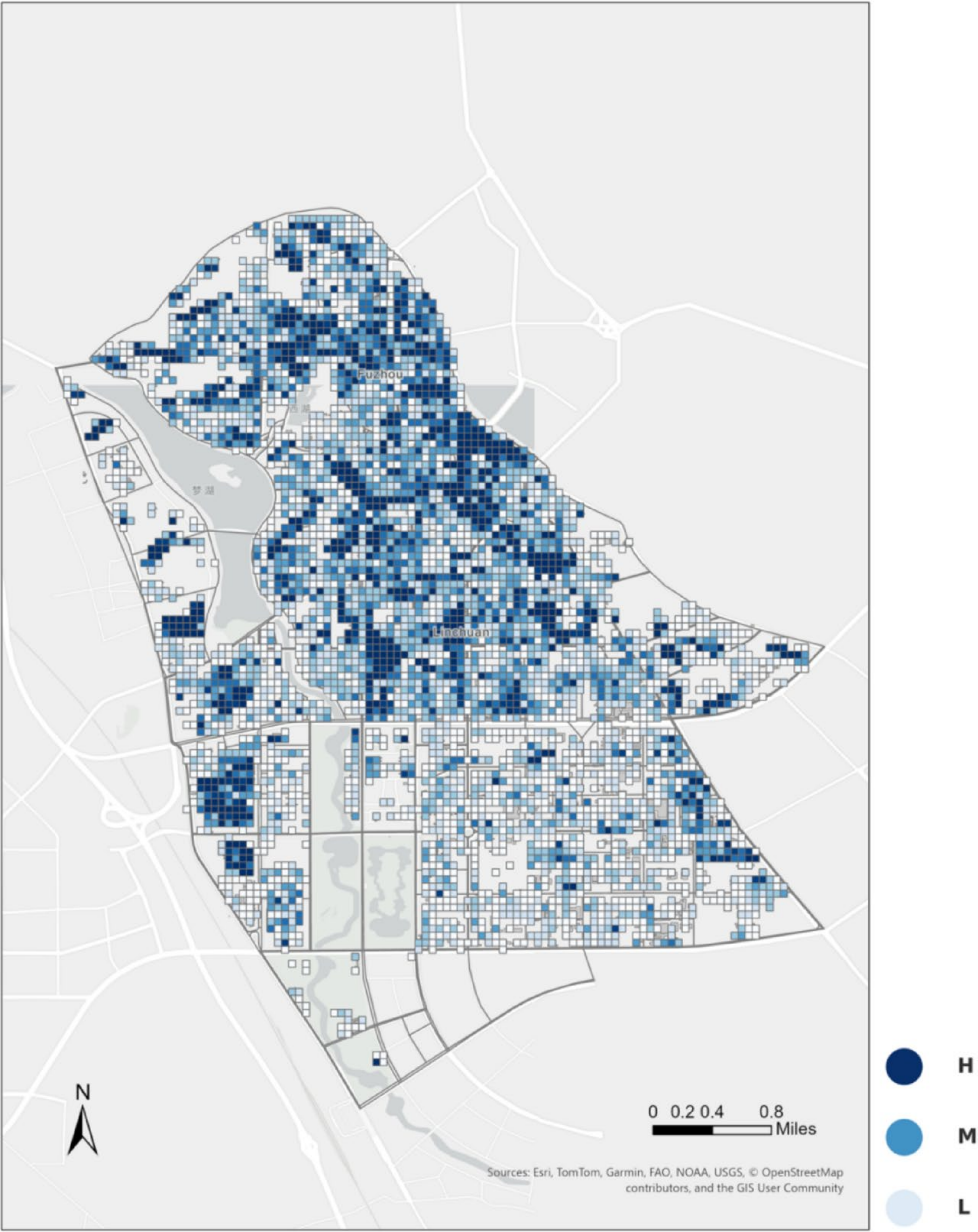
Composite walkability on a uniform grid. Values are mapped to a regular grid to reveal block-scale continuity; darker cells indicate higher scores. The grid view serves as a screening map to flag contiguous low-score patches and seams for follow-up checks. The map was created by the authors using ArcGIS Pro (version 3.4.2) (https://www.esri.com/en-us/arcgis/products/arcgis-pro/overview).

Specifically, the distribution pattern appears consistent with the Moran’s I result: high-score areas tend to cluster in central corridors—where physical connectivity, environmental comfort, and service capacity are simultaneously optimized—while low-score areas are more frequently located in peripheral neighborhoods, backstreets, and zones with poor walking environments, manifesting as contiguous “service deserts” (see Fig. 9)^40^.This spatial clustering not only corroborates the patterns observed in the preceding maps but also highlights that composite walkability is fundamentally shaped by the interplay between spatial configuration and environmental experience, rather than being an isolated point-based attribute^41^.

The observed configuration—H–H clusters near entrance-dense park belts and inner corridors, and L–L clusters toward peripheral blocks with fragmented local networks—aligns with monocentric-with-corridors morphologies reported in other large Chinese cities. By contrast, polycentric or hill–river interleaved settings tend to exhibit more dispersed H–H patches and mixed H–L seams near district boundaries. Against this backdrop, a Moran’s I around 0.39 can be read as moderate: strong enough to indicate non-random clustering, yet not dominated by a single corridor controlling citywide access. This reading suggests prioritizing spatially contiguous L–L patches and H–L seams (e.g., secondary entrances, missing-link sidewalks, safer crossings) where incremental, network-facing interventions may yield disproportionate gains.

## Discussion

### Key findings

This study developed an integrated walkability assessment model combining physical accessibility and visual perception dimensions. Using the central urban area of Fuzhou as a case study, we performed a multi-level spatial analysis spanning from street-level sampling points to park-scale units. Our analysis yields three principal insights.

First, replacing conventional radial buffers with network-based walking distance markedly refines walkability estimation: by tracing the true street topology and path complexity, this metric captures real walking conditions and avoids the systematic biases—such as neglected connectivity or impeded routes—that plague buffer methods^42^.

Second, a pronounced disjunction emerges between physical and perceived accessibility. Streets lying physically close to parks may score poorly in perception because sparse greenery, heavy spatial enclosure or degraded visual quality discourages elderly pedestrians, whereas more distant streets often attain high perceived accessibility when shaded, wide and visually open. Viewed through a person–environment fit lens, this pattern is a conclusion about mismatch: supportive visual cues reduce perceived demands and can offset distance, while adverse inner-backstreet cues elevate perceived demands even on short routes—hence access alone does not guarantee willingness and a coupled physical–perceptual lens is essential.

Third, composite scores cluster spatially: continuous high-access corridors align with well-integrated greenway–roadway bands, while the southern expansion zone, inner-block backstreets and urban fringes form contiguous “service deserts,” exposing a stark mismatch between green-infrastructure provision and the needs of local older residents. These inequities are consistent with broader findings in green equity research, where unequal access to urban greenery disproportionately affects vulnerable groups and has been linked to lower levels of well-being, delineating priority corridors for equity-oriented upgrades^43,44^.

### Differences from and contributions beyond previous studies

This study extends accessibility research on two fronts—evaluation perspective and spatial analysis scale.

First, it moves beyond the traditional single-dimensional lens, which typically privileges either physical distance or network connectivity, by coupling objective accessibility with subjective visual perception. This dual focus yields a fuller and more lifelike depiction of walking environments and foregrounds the needs of elderly pedestrians, thereby overcoming the constraints of distance-based assessments alone^45^. This dual focus is consistent with recent evidence across perception-based renewal, green-equity–well-being analyses, greenery–mobility links in compact cities, and street-view-based visual-enclosure studies on elders’ mental health^3,4,18,44^.

Second, it pivots from object-centred analyses that rely on simple buffers around parks or neighbourhoods to a path-experience-centred approach: street-level sampling points serve as the fundamental units, with integrated path networks and streetscape imagery nested from micro walking paths to park-scale service areas, forming a closed “path–experience–destination” loop. Such multi-scale coupling better reflects real-world walking contexts and captures the heterogeneous locational conditions that shape different parks’ catchments, offering sharper insights for spatial optimisation^46^.

Notably, despite these conceptual and methodological advances, the study corroborates earlier findings on the pivotal roles of green views, street continuity and barrier-free infrastructure, underscoring the enduring influence of key environmental factors on walkability.

### Research limitations

Despite the theoretical innovations and modeling strategies adopted in this study, several limitations remain that warrant improvement in future research:

First, the static and time-limited nature of the streetscape imagery. The street-view data used in this study were captured during a single period and are not temporally consistent across all locations. Some segments may be based on imagery from earlier years, while others are more recent, leading to uneven coverage. Moreover, seasonal differences in tree canopy, lighting, and pedestrian activity are not captured, and construction or redevelopment after the imagery date is unaccounted for. Such limitations may introduce bias, either overstating or understating the actual walking environment faced by older adults^47^.

Second, single-radius assumption and transferability. We use a fixed 1,200 m walking service radius as a policy-aligned simplification, yet older adults are heterogeneous in age, capacity, and mobility. Multi-source evidence indicates substantial intra-group differences in street age-friendliness and equity-relevant exposure constraints, supporting age-stratified thresholds, cohort-specific distance bands, and systematic sensitivity checks^43,48,49^. Looking ahead, travel-time–based service areas that integrate network impedance and temporal costs provide a more precise alternative to fixed radii^50^. Accordingly, external transfer of our results should involve local recalibration of parameters and perception weights; here we interpret radius-based outputs as pattern-oriented and report distance-threshold sensitivity (900 m, 1,500 m; Supplementary Table 1) rather than claiming plug-and-play generalizability^51^.

Third, the lack of empirical validation using real-world walking behavior. The present analysis is grounded in spatial modeling and visual metrics, but does not incorporate behavior-tracking data, such as GPS traces, mobile positioning, or park-entrance counts, nor field observations. As a result, the consistency—or potential discrepancies—between model predictions and actual walking behaviors of older adults remain unverified. This limitation confines the study’s conclusions to a theoretical and model-based level, highlighting the need for future validation through real-world behavioral data^52^.

Fourth, limits of perceptual calibration. The calibration draws on a small, locally recruited elderly panel and expert input, which constrains statistical power and may reflect local preferences. Future work should expand the sample and diversify participants.

### Future research directions

Future work should pivot from static appraisals to dynamic observations by incorporating time-stamped streetscape imagery, traffic flow and pedestrian-density feeds, thereby tracking temporal shifts in walking conditions and sharpening the currency of assessments; pair model outputs with GPS or wearable-derived traces of older pedestrians to ground predictions in real behaviour and iteratively tune the framework; Large-scale behavioral datasets, such as mobile phone positioning records, could be particularly valuable for directly validating predicted accessibility patterns and aligning model outputs with actual walking behaviors among elderly residents^53^.

Refine service radii to account for the pronounced heterogeneity of elderly cohorts—stratified by age, health status and mobility—so that scale and scope flex with need; the robustness of the framework should also be systematically tested across broader thresholds, weightings, and cultural contexts to enhance its external validity and transferability; and, crucially, embed the proposed spatial interventions in longitudinal, before-and-after trials to quantify their real-world impact on both environment and walking habits. Pursuing these strands in concert will strengthen the scientific rigour and practical utility of walkability evaluations, yielding more responsive guidance for evidence-based urban design^51,54,55^.

## Conclusion

This study focuses on the walkability of urban parks under the context of age-friendly cities, addressing the dual experiential needs of elderly pedestrians—namely, “being able to walk” and “willing to walk.” We proposed a multidimensional evaluation framework that integrates physical path resistance and streetscape visual perception. By constructing a street-level sampling network in the central urban area of Fuzhou, we integrated multiple data sources—including streetscape imagery, spatial networks, and population distribution—to conduct a full-scale spatial analysis ranging from point-level perception to path-based accessibility and park-level service units. The key conclusions are summarized as follows:

First, although physical accessibility serves as the foundational condition for park services, it alone is insufficient to fully explain the observed accessibility patterns and walking-related spatial outcomes of elderly pedestrians. Our findings reveal that some street segments, despite having smooth paths and short distances, lack attractiveness due to issues such as high visual enclosure and insufficient shading. This suggests that, for elderly pedestrians, spatial costs are not the sole explanatory factor, and that environmental perception plays a central role in shaping walking-related experiences and route evaluations.

Second, the visual perception dimension was clearly quantified in this study and demonstrated significant interactions with path accessibility. Through semantic segmentation and expert scoring, we extracted and integrated perceptual indicators such as green view index, obstruction rate, and sidewalk continuity to construct an accessibility scoring system that reflects the psychological responses of elderly pedestrians. The results show that areas with high perceptual scores generally correspond to higher composite accessibility scores, whereas regions with strong physical accessibility but low perceptual scores tend to have limited actual service effectiveness.

Third, the composite accessibility index reveals structural inequities in the provision of urban green space services. High-accessibility zones are concentrated along central axes and green corridors such as the Menghu Lake ecological belt, where path connectivity and landscape environments are well coordinated, exhibiting strong spatial clustering. In contrast, low-accessibility areas often overlap with neighborhoods facing significant population ageing, forming “service depressions” and highlighting the issue of “perceptual inequity” hidden beneath the surface of “formal spatial equity”^56^.

**Fig. 10:**
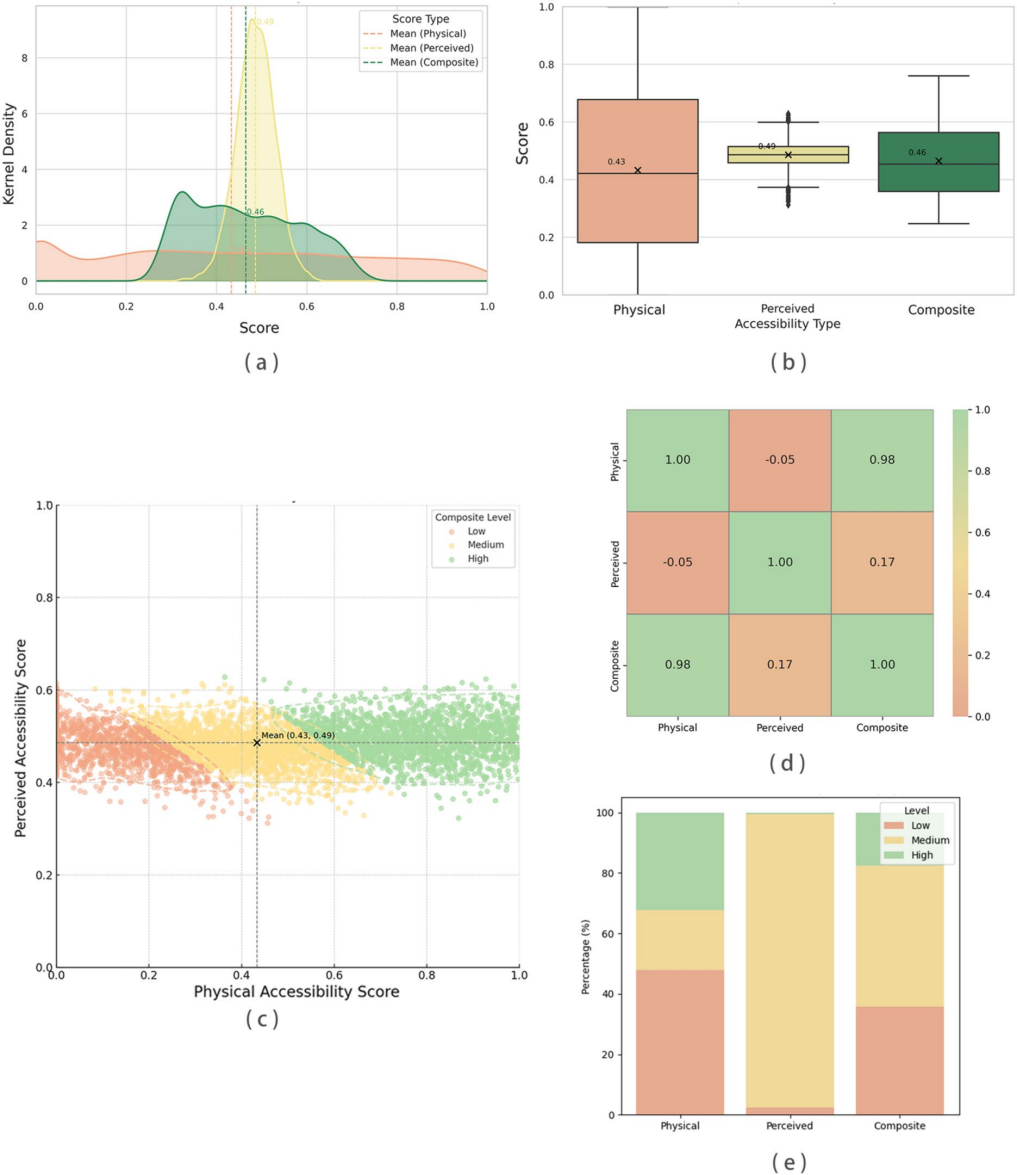
Distributional analysis of accessibility scores and internal relations among score types. (**a**) Kernel density estimation (KDE) plots of physical, perceived, and composite accessibility scores with vertical dashed lines indicating the mean values. (**b**) Boxplots of the three score types showing median, quartiles, and outliers. (**c**) Scatter plot of perceived vs. physical accessibility scores colored by composite score level (Low, Medium, High). (**d**) Heatmap matrix showing the Pearson correlation coefficients among the three score types. (**e**) Stacked bar chart illustrating the proportions of Low, Medium, and High levels within each accessibility type.

**Fig. 11:**
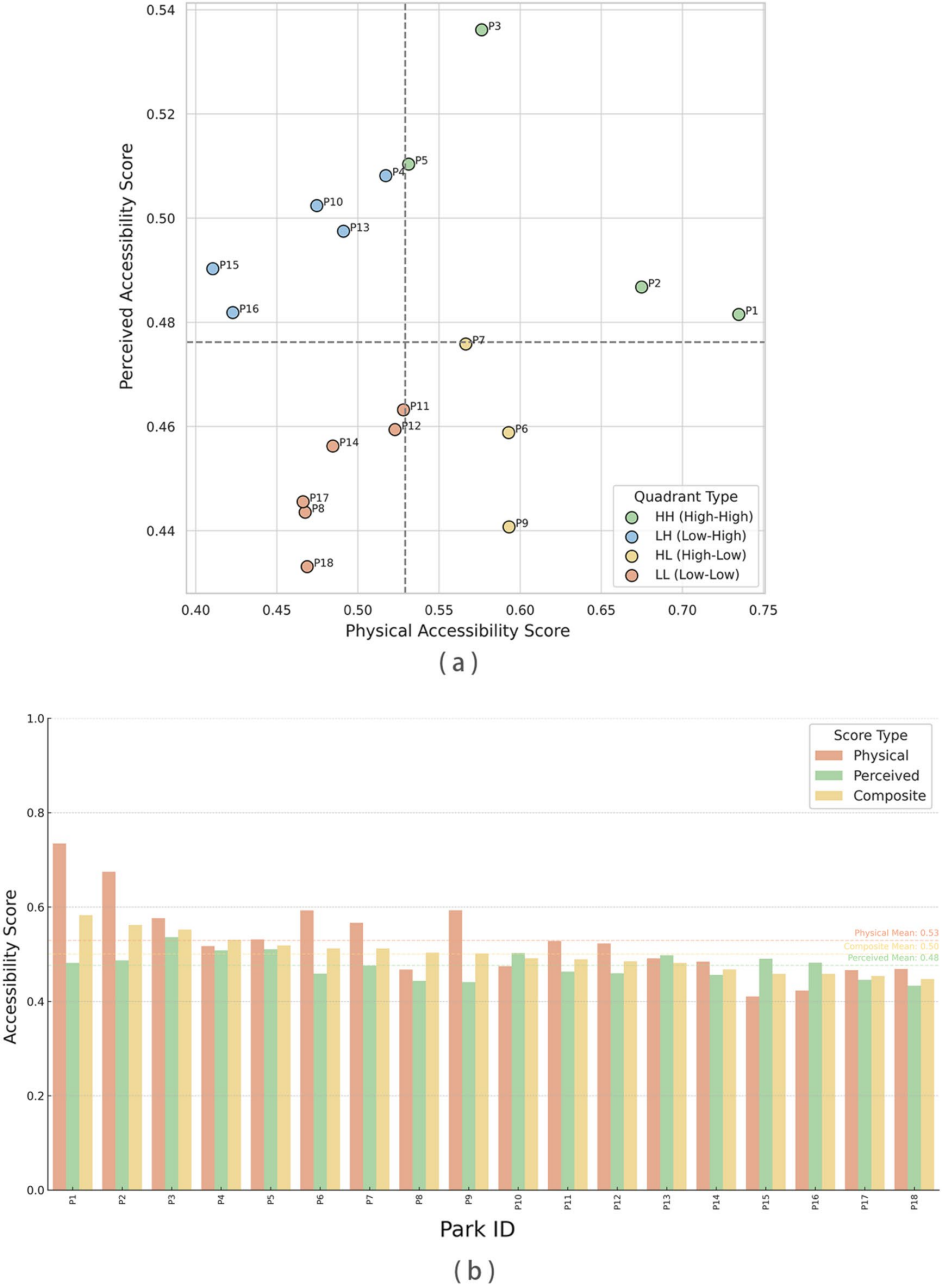
Comparison of park accessibility based on quadrant classification and score types. (**a**) Scatter plot showing the quadrant classification of 18 parks based on physical and perceived accessibility scores. Dashed lines represent the respective mean values used to delineate four quadrants: High-High (HH), High-Low (HL), Low–High (LH), and Low-Low (LL). Each dot corresponds to a park (P1–P18), colored by quadrant type, and ranked in descending order of composite accessibility. (**b**) Bar chart comparing physical, perceived, and composite accessibility scores of the 18 parks, ordered by composite score. Horizontal dashed lines indicate the mean score of each type.

Interventions should therefore be prioritized where person–environment misfit is most acute—pairing network repairs (safer crossings, missing-link sidewalks) with streetscape upgrades (shade, legible and continuous interfaces) to convert nominal coverage into effective use (Figs. 10, 11).

As a single-city case study in Fuzhou, these findings should be read as context-specific insights rather than universally generalizable results. Applying the framework elsewhere is feasible, but transferring the parameters and perception weights will require local recalibration and, where possible, validation with behavioral data.

## Supplementary Information

Below is the link to the electronic supplementary material.

Supplementary Material 1

Supplementary Material 2

Supplementary Material 3

Supplementary Material 4

Supplementary Material 5

## Data Availability

We understand the importance of data transparency and reproducibility in scientific research. This study utilizes multi-source spatial datasets, including OpenStreetMap street networks, demographic distributions, Google Street View imagery, and urban park boundaries. All data were obtained through legal and ethical means and processed using open-source tools and deep learning models. Due to platform restrictions, raw imagery cannot be publicly shared. However, the derived accessibility indicators, processed datasets, and analysis code are available from the corresponding author upon reasonable request. For data access inquiries, please contact Kaige Zheng.
